# The effect of forest-based health and wellness on the stress-relieve of middle-aged people

**DOI:** 10.3389/fpubh.2024.1366339

**Published:** 2024-05-07

**Authors:** Wei Quan, Shaona Yu, Qi Huang, Miaomiao Ying

**Affiliations:** ^1^Zhejiang College of Security Technology, Wenzhou Key Laboratory of Natural Disaster Remote Sensing Monitoring and Early Warning, Wenzhou, China; ^2^Wenzhou Vocational College of Science and Technology, Wenzhou Key Laboratory of Adding Carbon Sinks and Reducing Carbon Emissions of Agriculture, Forestry and Fishery Ecosystem, Wenzhou, China

**Keywords:** forest-based health and wellness, forest bath, middle-aged people, stress-relieve, total mood disturbance

## Abstract

In order to explore the impact of experience in forest-based health and wellness (FHW) on the stress of middle-aged people, 12 participants aged 35–39 were selected to conduct a 3-day/2-night study on FHW experience in Wencheng, Wenzhou. Huawei bracelets were used to monitor participants’ movement, pulse and blood pressure and their mood state was measured before and after the health care experience using the Profile of Mood States (POMS) scale. After the FHW experience, the lowest value of bracelet stress appeared on the second day of the experience for men and women. The total mood disturbance (TMD) decreased by 38.8 points on average, which significantly improved the positive mood and relieved the stress. The decompression effect of the FHW experience showed some variability among individuals. Furthermore, there were gender differences in alleviation of fatigue and puzzlement, which was greater for females than males.

## 1 Introduction

In China, FHW is based on high-quality forest resources and good forest environment. Guided by health theory and supported by the combination of traditional medicine and modern medicine, FHW has developed forest medical treatment, convalescence, rehabilitation, health care and health preservation, while also taking into account leisure, recreation, and vacation, etc. As such, FHW has the main functions of nourishing body, mind, temperament, wisdom, and morality, all of which are beneficial to human physical and mental health (1). It is similar in effect to forest therapist in Japan. Forest therapist stems from forest bathing, a short leisurely visit to a forest for the purpose of relaxation, called “Shinrin-yoku” in Japan (2).

As human society becomes increasingly urbanized, modern societies are subject to high levels of stress owing to the fast pace of life. People living in urban areas are prone to irritability and tiredness due to being in a crowded, unpleasant environment that is characterized by noisy traffic and unpleasant smells su6ch as automobile exhausts (3). Psychologically, stress refers to mental restraint and tension. The source of stress is external stimuli, such as tasks and challenges. Appropriate stress can be beneficial, for instance it can improve the efficiency of work and learning. However excessive stress can cause physical and mental suffering and even affect physical health.

According to the China National Bureau of Statistics, 35–60 years old is middle-aged. Middle-aged people are the predominant support of social labor force, family, and social responsibility which means middle-aged people tend to report more stress than any other age group (4). Various physiological and psychological diseases are caused by stress, thus stress greatly affects humans’ health and well-being (5, 6). Forest bathing or forest therapy refers to immersing oneself in nature and experiencing a forest’s atmosphere in order to improve mental and physical health (7–11). This therapeutic experience engages all five senses, with activities such as walking or simply being in the forest, which can effectively alleviate stress and confer numerous health benefits (12). As a preventive and alternative treatment approach, forest therapy has gained prominence as a means to manage stress and promote overall health and well-being, stemming from the restorative effects of spending time in a green, healthy environment (13).

Wencheng County is located in the mountainous area of southern Zhejiang Province, China (119°46′–120°15′E, 27°34′–57°59’N). Wencheng County boundary belongs to subtropical marine monsoon climate area, the forest coverage rate is 71.5%, its annual average temperature is 14–18.5°C, and it has a perennial frost-free period of 285 days. The diet and accommodation within the FHW experience study were arranged in Yueman FHW base in Wencheng County, which is a demonstration FHW base in Wenzhou City. This study will investigate the effects of FHW experience on the decompression and mood regulation in a middle-aged sample through monitoring the dynamic changes of exercise parameters, routine physiological indicators, and stress of the participants. Evaluating the potential psychological and physiological benefits of the short FHW program could help to further develop this type of program, provide proof of its effectiveness, and encourage the public to enter the forest, thus promoting the development of FHW industry and improving national health.

## 2 Research methods

### 2.1 Participants

Five males and seven females, aged 35–39 years (mean age = 37.1) were selected as participants. Height range 151–184.5 cm (mean height = 168.6 cm). The body weight ranged from 45.3 to 87 kg (mean weight = 65 kg). Body Mass Index (BMI) ranged from 19.1 to 25.6 (mean BMI = 22.8). See Table 1 for details. The participants all signed the commitment and their family members also signed the informed consent. During the study period, the participants had no disease characteristics and did not take any drugs.

**Table 1:** Participant base data.

Sex	Age (year)	Height (cm)	Weight (kg)	BMI
Male	37–39	169.5–184.5	61.6–87	19.1–25.6
Female	35–39	151–172.5	45.3–72.9	19.4–24.5
Mean	37.1	168.5	65.0	22.8

### 2.2 Experimental procedure

The participants participated in a 3-day/2-night FHW experience in Wencheng (July 6th to July 8th, 2019). Each person wore Huawei Glory 4 bracelets. A forest walk was arranged for each day, along with other activities such as enjoying lotus flowers, visiting Liu Bowen’s hometown, and a forest hot spring bath. Blood pressure, pulse, and blood oxygen were measured using a LKang physical examination machine.

The POMS scale consists of 40 adjectives describing different emotional states, including seven different emotions: tension, anger, fatigue, depression, panic, vigor, and self-esteem. It is widely used in stress level detection and stress management. The total score of mood scale is TMD = negative emotion (tension + anger + fatigue + depression + panic) − positive emotion (vigor + self-esteem) + 100. The Profile of Mood States (POMS) scale was administered on July 1st before the experience and on July 8th after the experience.

Huawei B5 bracelets were used for the stress test, which has passed the authoritative test certification of Institute of Psychology, Chinese Academy of Sciences. Stress was administered 4 times: in the evening of July 6th, the morning of July 7th, the evening of July 7th, and the morning of July 8th, from 7:30 to 8:30 in the morning and evening.

The study was approved by Dian Diagnostic medical ethics committee, and the procedures were in accordance with the Helsinki Declaration of 1975 as revised in 1983.

### 2.3 Statistical analysis

EXCEL 2003 was used for data calculation and mapping. The differences of mood state before and after the experience and the difference of stress values at different stages were tested by paired *t*-test with SPSS Statistics 17.

## 3 Results

### 3.1 Sports situation

From July 6th to July 8th, the average daily steps of 12 participants reached 13227.9 steps, the highest of which was July 6th, with some individuals exceeding 20,000 steps. The mean distance over the three days was 9.8 km, and the daily *per capita* calorie consumption was 469.3 kcal. The intensity of 3-day exercise decreased slightly, as shown in Table 2.

**Table 2:** Sports profiles of participants.

Time		Steps (mean ± SD)	Distance/km (mean ± SD)	Heat/Kcal (mean ± SD)
July 6th	Average	15217.4 ± 4142.2	11.4 ± 3.3	529.8 ± 228.8
	Male	18712.8 ± 2001.6	14.3 ± 1.9	727.4 ± 53.4
	Female	12720.7 ± 3366.3	9.4 ± 2.4	388.6 ± 195.6
July 7th	Average	14462.0 ± 4759.7	10.9 ± 4.2	502.2 ± 342.3
	Male	18773.8 ± 5102.4	14.6 ± 5.1	815.5 ± 359.7
	Female	12220.7 ± 2553.8	9.0 ± 1.8	354.9 ± 201.5
July 8th	Average	10004.0 ± 3970.2	7.16 ± 2.7	375.8 ± 206.7
	Male	12486.6 ± 3810.6	8.9 ± 2.6	542.0 ± 130.7
	Female	8231.1 ± 3227.1	6.0 ± 2.1	257.1 ± 165.9
Average		13227.9 ± 4726.2	9.8 ± 3.8	469.3 ± 263.7

### 3.2 Effect on blood pressure, blood oxygen, and pulse

The Chinese Guidelines for the Prevention and Treatment of Hypertension (2010 Revised Edition) stated that the normal blood pressure of adults in China is <120 mmgh in systolic blood pressure and < 80 mmgh in diastolic blood pressure. During the FHW experience, the mean diastolic and systolic blood pressures of 12 participants were within the normal range at all four recordings. As depicted in Table 3, both diastolic and systolic blood pressures exhibited an increase on the evening of July 7th relative to the previous evening, the 6th. Conversely, a marked decrease in both pressure readings was observed on the morning of July 8th in comparison to the morning of July 7th.

**Table 3:** Blood pressure of participants.

Time	Diastolic (mmgh)			Systolic (mmgh)		
	Average	Male	Female	Average	Male	Female
July 6th evening	71.3 ± 12.6	72.6 ± 13.5	70.4 ± 12.9	101.3 ± 18.6	112.0 ± 17.8	93.7 ± 16.1
July 7th morning	71.4 ± 8.1	75.4 ± 8.4	68.6 ± 7.1	110.1 ± 10.6	117.6 ± 11.4	104.7 ± 6.2
July 7th evening	72.6 ± 8.9	77.0 ± 10.1	69.6 ± 6.9	104.4 ± 12.6	110.4 ± 17.1	100.1 ± 6.9
July 8th morning	66.3 ± 11.0	74.4 ± 9.0	60.6 ± 8.6	93.4 ± 18.1	108.6 ± 10.6	82.6 ± 14.0

When at rest, the normal heart rate of adults is 60–100 beats per minute with the ideal heart rate being 55–70 beats per minute. The heart rate of females is consistently faster than that of males of the same age (14). During the FHW experience, the participants’ pulse was measured four times. Although pulse rates showed a small range of fluctuation, they were within the normal range (see Table 4).

**Table 4:** Pulse and blood oxygen status of participants.

Time	Pulse (beats/min)			Blood oxygen (%)		
	Average	Male	Female	Average	Male	Female
July 6th evening	76.4 ± 9.9	74.0 ± 14.2	78.1 ± 6.0	0.97 ± 0.0	0.96 ± 0.0	0.97 ± 0.0
July 7th morning	77.4 ± 9.7	77.6 ± 10.0	77.3 ± 10.3	0.97 ± 0.0	0.96 ± 0.0	0.97 ± 0.0
July 7th evening	79.0 ± 10.5	74.0 ± 10.4	82.6 ± 9.7	0.97 ± 0.0	0.96 ± 0.0	0.97 ± 0.0
July 8th morning	76.2 ± 11.5	74.8 ± 15.1	77.1 ± 9.3	0.97 ± 0.0	0.97 ± 0.0	0.97 ± 0.0

It is generally believed that the normal blood oxygen level should not be less than 94% and that less than 94% is considered to be insufficient oxygen supply. The higher the oxygen content in the blood, the better the metabolism (15). During the FHW experience, the blood pressure saturation of the 12 participants was tested on four occasions. Oxygen content was 96% or above, in a normal and good state, with rates of 100% in some individuals. The blood pressure saturation showed a stable trend and slightly increased.

### 3.3 Effects on the state of mind

The higher TMD, the greater the negative mood. On the contrary, the more positive of the mind state (16). Compared to before the FHW experience the scores of negative emotions (tension, anger, fatigue, depression and panic) decreased, while the scores of positive emotions (vigor and self-esteem) increased in the post-experience measurements (see Table 5). The mean TMD decreased by 38.6 points (male TMD by 33.6 points and female TMD by 42.4 points). FHW experience has a more obvious effect on alleviating female stress.

**Table 5:** Assessment of mood state.

Sex	Stage	Tension	Anger	Fatigue	Depression	Vigor	Confusion	Self-esteem	TMD
Total	Before	8.3 ± 3.8	8.1 ± 5.1	8.1 ± 2.9	5.8 ± 3.1	10.3 ± 3.5	6.9 ± 3.1	8.1 ± 1.9	118.8 ± 15.3
	After	1.4 ± 1.3^**^	1.0 ± 1.8^**^	0.8 ± 1.2^**^	0.5 ± 1.2^**^	15.0 ± 4.1^**^	2.0 ± 2.3^**^	10.7 ± 2.9^**^	80.0 ± 9.4^**^
Male	Before	7.0 ± 2.8	9.0 ± 5.1	6.6 ± 1.8	4.4 ± 2.6	10.6 ± 4.5	5.4 ± 1.7	8.2 ± 2.3	113.6 ± 16.5
	After	1.2 ± 1.1^**^	1.0 ± 1.0^**^	0.6 ± 0.9^**^	0.2 ± 0.4^**^	15.0 ± 2.8^**^	1.8 ± 1.1^**^	9.8 ± 1.9^**^	80.0 ± 6.7^**^
Female	Before	9.1 ± 4.4	7.4 ± 5.4	9.1 ± 3.2	6.7 ± 3.2	10.0 ± 3.1	8.0 ± 3.6	8.0 ± 1.8	122.4 ± 14.5
	After	1.6 ± 1.5^**^	1.0 ± 2.2^**^	0.9 ± 1.5^**^	0.7 ± 1.5^**^	15.0 ± 5.0^**^	2.1 ± 3.0^**^	11.3 ± 3.4^**^	80.0 ± 11.4^**^

A statistically significant difference is observed in the indicators post-FHW experience when compared to pre-FHW levels, with a *p*-value of less than 0.01 (**p < 0.01).

There were significant differences in tension, anger, fatigue, depression, vigor, panic, self-esteem and TMD before and after FHW experience (*p* < 0.01), the fatigue index changed the most among all indicators. FHW experience activities significantly improved the mood of middle-aged participants. This is completely consistent with the previous research conclusions of many scholars (17–19). FHW of middle-aged people has the same effect on improving emotions of young and older adults, and the effect of reducing fatigue and confusion of female is better than that of male.

### 3.4 Effect on stress

Stress state is controlled by the autonomic nervous system, in which the increase of sympathetic activity will increase the level of stress, while the increase of parasympathetic activity will reduce the level of stress. The state of the autonomic nervous system can be assessed by heart rate variability, thus heart rate variability is a good indicator of stress. Huawei stress detection technology uses HRV data, combined with a corresponding stress calculation model and stress sensors to estimate the stress state and degree of users.

**Figure 1:**
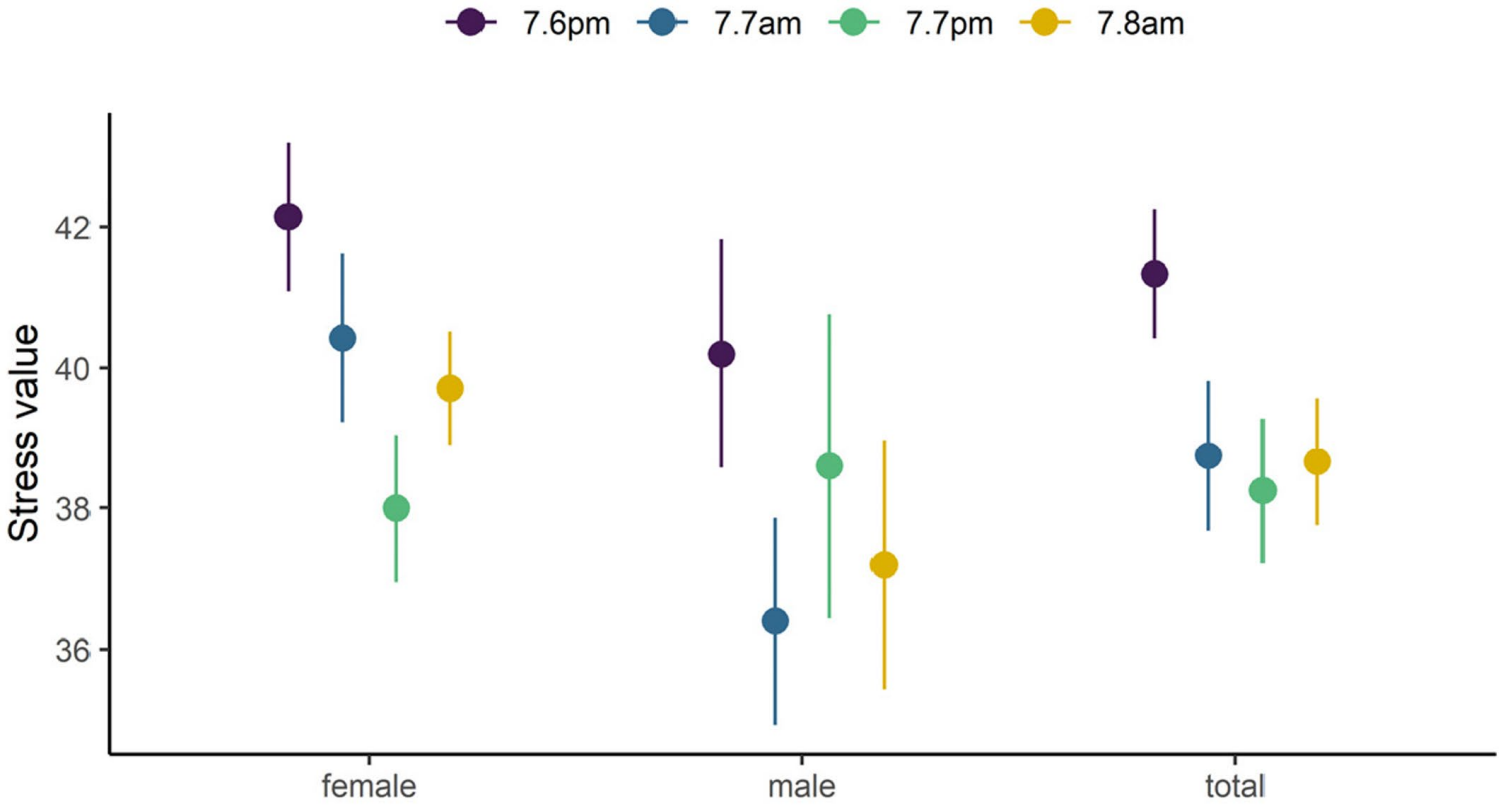
Stress values at different stages.

During the FHW experience, the participants’ mean stress value reached the highest (41.3) on the first day and the lowest (38.3) on the second night of the experience, showing a U-shaped trend. For males, the highest stress value was 40.2 on the first day and the lowest value was 36.4 on the second day, showing a wave-like trend. For females, the highest stress level was 42.1 on the first day and the lowest was 38.0 on the second night, showing a U-shaped trend. Some experienced a decrease of 8–9 stress values within 24 h. During the FHW experience, there were some differences between male and female stress values, for instance the effect of decompression was faster for males. The lowest value both appeared on the second day of the experience, but there was no significant difference in the stress values of the participants at different stages (*p* > 0.05) (see Figure 1).

## 4 Discussion

It has been reported that a higher ambient temperature reduces blood pressure, whereas a lower ambient temperature raises blood pressure (20–23). Several studies have determined that participants walking in a forest environment have lower pulse rates and diastolic and systolic blood pressures than in urban settings (17, 24–31). However there was no significant difference in blood pressure between the forest and urban area walking groups because of the big difference in ambient temperature between the forest (lower temperature) and urban (higher temperature) environments (2). Forest bathing significantly reduces blood pressure by reducing sympathetic nerve activity and urinary adrenaline, noradrenaline, and dopamine levels (2). In this study, the diastolic and systolic blood pressures of the participants showed a trend of first rising and then decreasing. The initial elevation of blood pressure may have been caused by the lower forest temperature and then the forest environment caused the later decrease.

The POMS test is a well-accepted quantitative means of evaluating mood, widely used in psychological investigations (3). The POMS measurements in the current study confirm previous findings that forest environments can relieve human psychological tension, depression, anger, fatigue, and confusion (27), suggesting that the subjects were physiologically relaxed during the forest bathing trips (17, 27, 30, 32–40). Some studies have also reported that forest environments can lead to improvements in other psychological responses, including anxiety and depression (29, 41–45). Kasetani et al. reported that a relationship exists between the POMS score and the physical environmental factors, such as relative illumination, relative humidity, atmospheric pressure, etc. (46). Studies have found that individuals with high blood pressure or high stress levels are more likely to exhibit improvement after forest bathing than healthy individuals (32, 44). It is important that the improvement in POMS score for those with depressive tendencies was much greater than for the non-depressive tendencies after forest bathing (47). Improvements observed in depressive symptoms and psychosocial functions may be linked to the possible activation of neural networks through positive emotional reflection (48), and improved social cognitive functions through the stimulation of mirror neurons, leading to a better understanding of the intended actions and emotions of others (49).

Previous studies have shown that compared with exposure to urban settings, exposure to forest environments results in higher parasympathetic and lower sympathetic nervous activity (10, 17, 27, 43). Both epinephrine levels and cortisol levels have been shown to decrease after a forest bathing trip suggesting forest bathing trip could reduce the stress level of Chronic Obstructive Pulmonary Disease (COPD) patients (50). From the viewpoint of attention restorative theory (51), these results strongly support that the forest is a good restorative environment for human beings (27). These studies have provided some evidence that living in a forest environment, even for a short time, exerts benefits on human health (3). Evaluating the effects of the short forest bathing program on psychological and physiological benefits could help to develop this type of program, provide proof of its effectiveness, and encourage the public to connect with nature (19). In addition, forest bathing may represent a potential medical intervention in several pathologies, including inflammation and cardiovascular and nervous conditions (3).

## 5 Conclusion

After the forest recuperation experience, both males and females demonstrated significantly reduced negative emotions, significantly increased positive emotions, and a decreased stress value on the second day of the experience. Both subjective and objective physiological evaluation indicators showed that the forest recuperation experience activities relieved stress. FHW did have a relaxing effect, promoting health, and alleviating fatigue and sleepiness of females more so than males. What factors improve mood state? Quiet atmosphere, beautiful scenery, mild climate, fresh air, and other forest environments can increase parasympathetic activity and reduce sympathetic activity (27). Because of the particularity of environments and individual differences, the effect of forest environment health care and recuperation varies from person to person (52). The forest walk and other activities during the FHW experience may also have a beneficial impact on the emotional state. The improvement of the participants’ emotions is the result of the above factors and other comprehensive factors. Due to the short duration of this study and the small number of participants, the exact conclusion of the positive effects of FHW requires further research, which should involve a larger cohort of participants and extend the period of exposure to FHW. Recognizing FHW as a multidisciplinary field, involves multiple fields such as forestry, medicine, psychology, management, and education, and requires interdisciplinary cooperation and participation (13).

## Data availability statement

The original contributions presented in the study are included in the article/supplementary material, further inquiries can be directed to the corresponding author.

## Ethics statement

The studies involving humans were approved by the ethics committee of Dian Diagnostic. The studies were conducted in accordance with the local legislation and institutional requirements. The participants provided their written informed consent to participate in this study.

## Author contributions

WQ: Writing – original draft, Writing – review & editing, Data curation, Investigation, Methodology, Project administration. SY: Writing – review & editing, Data curation, Investigation. QH: Conceptualization, Investigation, Resources, Writing – review & editing, Software. MY: Funding acquisition, Writing – review & editing, Project administration.
